# Clinical service evaluation of the feasibility and reproducibility of novel artificial intelligence based-echocardiographic quantification of global longitudinal strain and left ventricular ejection fraction in trastuzumab-treated patients

**DOI:** 10.3389/fcvm.2023.1250311

**Published:** 2023-11-13

**Authors:** J. Jiang, B. Liu, Y. W. Li, S. S. Hothi

**Affiliations:** ^1^ Heart and Lung Centre, New Cross Hospital, Royal Wolverhampton NHS Trust, Wolverhampton, United Kingdom; ^2^ Department of Cardiology, Manchester University NHS Foundation Trust, Manchester, United Kingdom; ^3^ Institute of Cardiovascular Sciences, University of Birmingham, Birmingham, United Kingdom; ^4^ Department of Anaesthesia, New Cross Hospital, Royal Wolverhampton NHS Trust, Wolverhampton, United Kingdom; ^5^ Research Centre for Health and Life Sciences, Coventry University, Coventry, United Kingdom

**Keywords:** cardio-oncology, trastuzumab, cardiotoxicity, artificial intelligence, strain, echocardiography

## Abstract

**Introduction:**

Cardiotoxicity is a potential prognostically important complication of certain chemotherapeutic agents that may result in preclinical or overt clinical heart failure. In some cases, chemotherapy must be withheld when left ventricular (LV) systolic function becomes significantly impaired, to protect cardiac function at the expense of a change in the oncological treatment plan, leading to associated changes in oncological prognosis. Accordingly, patients receiving potentially cardiotoxic chemotherapy undergo routine surveillance before, during and following completion of therapy, usually with transthoracic echocardiography (TTE). Recent advancements in AI-based cardiac imaging reveal areas of promise but key challenges remain. There are ongoing questions as to whether the ability of AI to detect subtle changes in individual patients is at a level equivalent to manual analysis. This raises the question as to whether AI-based left ventricular strain analysis could provide a potential solution to left ventricular systolic function analysis in a manner equivocal to or superior to conventional assessment, in a real-world clinical service. AI based automated analyses may represent a potential solution for addressing the pressure of increasing echocardiographic demands within limited service-capacity healthcare systems, in addition to facilitating more accurate diagnoses.

**Methods:**

This clinical service evaluation aims to establish whether AI-automated analysis compared to conventional methods (1) is a feasible method for assessing LV-GLS and LVEF, (2) yields moderate to good correlation between the two approaches, and (3) would lead to different clinical recommendations with serial surveillance in a real-world clinical population.

**Results and Discussion:**

We observed a moderate correlation (*r* = 0.541) in GLS between AI automated assessment compared to conventional methods. The LVEF quantification between methods demonstrated a strong correlation (*r* = 0.895). AI-generated GLS and LVEF values compared reasonably well with conventional methods, demonstrating a similar temporal pattern throughout echocardiographic surveillance. The apical-three chamber view demonstrated the lowest correlation (*r* = 0.423) and revealed to be least successful for acquisition of GLS and LVEF. Compared to conventional methodology, AI-automated analysis has a significantly lower feasibility rate, demonstrating a success rate of 14% (GLS) and 51% (LVEF).

## Introduction

Cardiotoxicity is a significant, potential complication of certain chemotherapeutic agents that can lead to either preclinical or overt heart failure. In some cases, chemotherapy must be withheld when cardiac function, primarily left ventricular (LV) systolic function, becomes significantly impaired to protect cardiac function at the expense of a change in the oncological treatment plan and associated changes in prognosis (1). Accordingly, patients receiving potentially cardiotoxic chemotherapy are recommended to undergo routine surveillance before, during and following completion of therapy, usually with transthoracic echocardiography (TTE). Transthoracic echocardiography is a well-established and widely available imaging modality with an important role in determining cardiac structure and function. To date, it remains the preferred technique for assessing the development, progression and regression of cardiotoxicity among oncology patients undergoing cardiac surveillance (2).

Echocardiographic indices such as left ventricular ejection fraction (LVEF) by Simpson's Biplane method has traditionally been used to assess changes in LV systolic function. However, in the modern era of speckle tracking echocardiography (STE), strain quantification has rapidly evolved into a valuable tool for the early detection of cardiotoxicity during oncological therapy and has since been incorporated into international guidance (3, 4).

Until now, global longitudinal strain (GLS) has been the most studied strain parameter with the largest body of literature supporting its diagnostic and prognostic value (5, 6). One early study evaluated eighty-one females with newly diagnosed HER2 + breast cancer for early alterations of myocardial strain during treatment with anthracycline and/or trastuzumab. Patients received three-monthly surveillance throughout the course of a fifteen-month study period. A reduction in LVEF was observed in the overall cohort (64 ± 5% to 59 ± 6%; *p* < 0.0001); twenty-six patients [32%, (22%–43%)] developed cardiotoxicity, and of these patients, 5 [6%, (2%–14%)] developed symptoms of heart failure (HF). Significant LVEF reduction (≥8%) was detected in 15% of patients that developed subsequent cardiotoxicity, whereas upon the application of strain analysis, the incidence rate increased to 78%. Among the patients that later developed HF, all had a reported GLS of less than −19% (7).

While strain quantification with speckle tracking echocardiography represents a sensitive method for assessing LV function, this postprocessing analysis remains laborious, time-consuming and is subject to significant inter- and intra-observer variability, related to reproducibility of contouring cardiac structure by manual and even semi-automated contouring. In recent years, the emergence of artificial intelligence (AI) in echocardiography has generated much interest among the cardiac imaging community. The technology is rapidly evolving but is yet to be widely adopted into clinical practice. Recent evidence has revealed promising findings, demonstrating that the application of AI enables data analysis free from human operator bias, accelerated workflow and quantification, along with high feasibility rate in the absence of operator input. One multicentre study which assessed LVEF and longitudinal strain using visual, manual and fully AI-automated-methods (TomTec-Arena 1.2, TomTec Imaging Systems) reported a high feasibility (98%) of AI-automated assessment (8). Good correlation and levels of agreement were observed between manual and automated assessment (ICC: 0.83; bias: 0.7%; 95% CI: 0.1%–1.3%). Expectedly, bias and levels of agreement were wider when visual assessments were compared. A key advantage of automated LVEF and LV-GLS compared to manual and visual assessment was the absence of inter-measurement variability on repeated assessments with the AI method able to identify the same patterns each time. Finally, beat to-beat variability was 0.96 ± 3.52% for automated LVEF, 2.7 ± 8.16% for manual LVEF, 0.19 ± 1.31% for automated GLS, and 1.09 ± 3.29% for manual GLS (8).

In support of these findings is another recent trial by Salte et al., which reported good correlation (*R* = 0.93, *p* < 0.001) and low bias of −1.4 ± 0.3% (*p* < 0.01) with an estimated level of agreement (LOA) of ±3.7% when comparing AI-automated vs. conventional methodology (EchoPAC v.202, GE), suggesting that the application of AI is potentially comparable to human expert performance using conventional methodology (9).

While AI-based cardiac imaging analysis appear promising, there are areas that require further assessment. AI-automated analysis must be able to perform at least as well as established methodologies to detect subtle changes in left ventricular function, whether LVEF or GLS. Hence, further research is needed to fully establish the vulnerability of automated image processing networks. Furthermore, this automated approach relies upon a large training dataset to implicitly learn features of the heart relevant to segmentation which is resource intensive, demands close clinical supervision and raises potential ethical and privacy concerns.

If AI-automated analysis of LV function can be demonstrated to be equivocal to or superior to conventional methods within a real-world clinical service, then it may represent a potential solution for the challenges of limited clinical service capacity by reducing the pressures of increasing echocardiographic demands, in addition to facilitating more accurate diagnoses. This clinical service evaluation aims to establish whether AI-automated analysis is: (1) a feasible method for assessing LV-GLS and LVEF, (2) correlates well with conventional methods, and (3) whether AI analysis would lead to different clinical recommendations during serial surveillance in a real-world clinical population.

## Materials and methods

### Patient population

This single-centre audit and service evaluation retrospectively reviewed all HER2 + breast cancer patients that underwent TTE surveillance and trastuzumab therapy between January 2019 and October 2022 at the Royal Wolverhampton NHS Trust (UK) and assessed the evaluation of cardiac function against international cardio-oncology guidance (Audit/Service evaluation number 5918, Royal Wolverhampton NHS Trust, UK). Informed consent was not required due to the retrospective nature of the clinical audit and evaluation. Patients undergoing combination therapy including anthracycline were excluded from the study. Patients with atrial fibrillation or other form of arrhythmias during the echocardiographic studies were also excluded. To reflect real-world patient population and feasibility, patients with partially suboptimal endocardial border definition were not excluded. Clinical characteristics of our cohort were collected from the image reporting system and hospital records and are summarised in Table 1.

**Table 1:** Demographic and clinical characteristics of the patient population.

Age (years)	59 ± 13		
Gender	140 Female	2 Male	
ECG and HR (bpm)	142 SR 79 ± 13		
Height (cm)	163 ± 7.5		
Weight (kg)	76 ± 18		
BMI (kg/m^2^)	28.7 ± 6.4		
BSA (m^2^)	1.85 ± 0.2		
Blood pressure (BP)	Systolic BP 137 ± 26 mmHg	Diastolic BP 80 ± 14 mmHg	
Cancer type	119 BC	18 GC	5 OC

Data are expressed as mean ± standard deviation.

ECG, electrocardiogram; HR, heart rate; SR, sinus rhythm; AF, atrial fibrillation; BMI, body mass index; BSA, body surface area; BC, breast cancer; GC, gastric cancer; OC, oesophageal cancer.

### Echocardiographic imaging protocol and analysis

648 TTE studies acquired from 142 oncology patients that received trastuzumab echocardiographic surveillance between 2019 and 2022 were retrospectively evaluated. All echocardiographic studies within our British Society of Echocardiography (BSE) accredited imaging laboratory were comprehensive studies which complied with BSE cardio-oncology guidelines. Echo imaging was performed by BSE accredited echocardiographers using commercial equipment (Affiniti, EPIQ and iE33, Phillips Medical Systems, Andover, Massachusetts, USA).

### Assessment of GLS and LVEF

AI-automated and conventionally measured GLS and LVEF were assessed from standard apical four- (A4C), three- (A3C), and two-chamber (A2C) cine loops in accordance with BSE guidance.

AI-automated assessments (GLS and LVEF) were performed on individual echocardiographic studies using an AI-based platform (Ultromics EchoGo Core, Oxford, UK). The investigators submitted individual clinical studies required for analysis from the local hospital archiving system to the AI pipeline (Ultromics SaaS). Individual views are identified and classified with the existing convolutional neural network (CNN) model and subsequently processed by a U-Net based architecture for view-specific LV contouring, myocardial segmentation, and myocardial motion tracking to compute GLS and LVEF in the absence of manual adjustments (10).

Conventional GLS assessment was performed in a semi-automated fashion from the apical four-, three- and two-chamber LV-focused cine images in dedicated conventional software (QLab, version 15.5, Philips Medical Systems). Upon detection of the endocardial border, the software automatically established a region of interest (ROI) and calculated the strain values of the selected view. The BSE-accredited or similarly experienced operator manually adjusted the ROI to optimise tracking if deemed necessary and strain values were recalculated to reflect this adjustment. Where image quality was insufficient to permit strain assessment of all three views, then a global strain value could not be calculated. Conventional LVEF was manually performed using the Simpson's biplane method of discs (Modified Simpson's rule) for LV volumes and LVEF calculation. End-diastole was defined as the frame following mitral valve closure or the frame in which the cardiac dimension is largest, in preference to the onset of the QRS. End-systole was defined as the frame preceding mitral valve opening or the time in the cardiac cycle in which the cardiac dimension is smallest, respectively. This protocol was performed using the LV-focused A4C and A2C views.

### Statistical analysis

Continuous variables were expressed as mean ± standard deviation and categorical variables were presented as *n* (%). Linear regression analysis was performed to evaluate the relationship between GLS and LVEF when assessed by either conventional or AI-automated methods. Bland-Altman analysis was used to assess the levels of agreement and quantify systemic differences between assessments. Comparison of mean values between the automated and conventional groups were performed using the paired sample student *t*-test. Analysis of variance (ANOVA) was used to compare the means of three of more groups. For all statistical tests performed, a *p*-value less than 0.05 was regarded as statistically significant. Statistical analyses were performed using IBM SPSS Statistics version 29 (New York, USA).

## Results

### Subject characteristics

The patient cohort included 142 patients which had undergone a total of 648 echocardiographic studies as part of their oncological therapy cardiac surveillance. The population comprised 140 females (99%), with mean age 59 ± 13 years (range 28–89 years). Oncological diagnoses predominantly comprised breast cancer (84%), but also included gastric (13%) and oesophageal cancer (3%). Patient demographic and clinical characteristics are summarised in Table 1.

### Technical feasibility of AI-based compared to conventional assessment in GLS and LVEF

**Figure 1:**
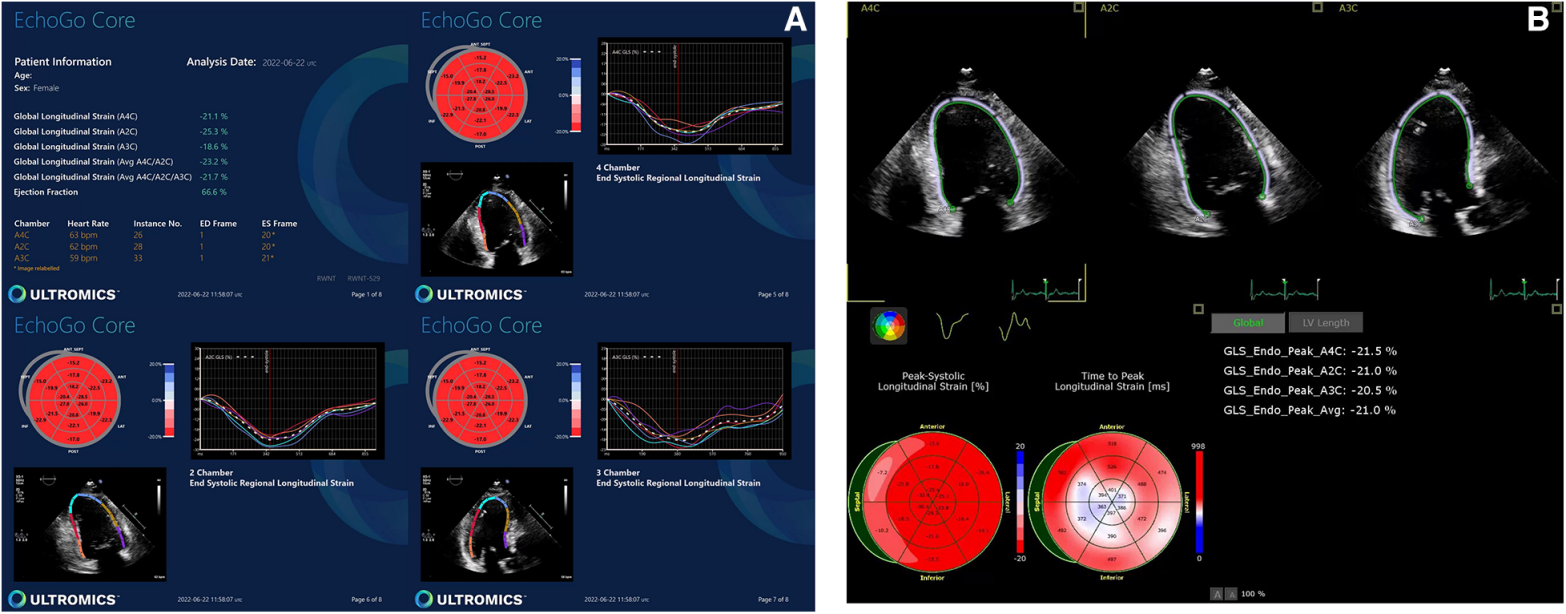
Normal GLS data yielded by (**A**) AI-based and (**B**) conventional semi-automated strain analysis.

**Figure 2:**
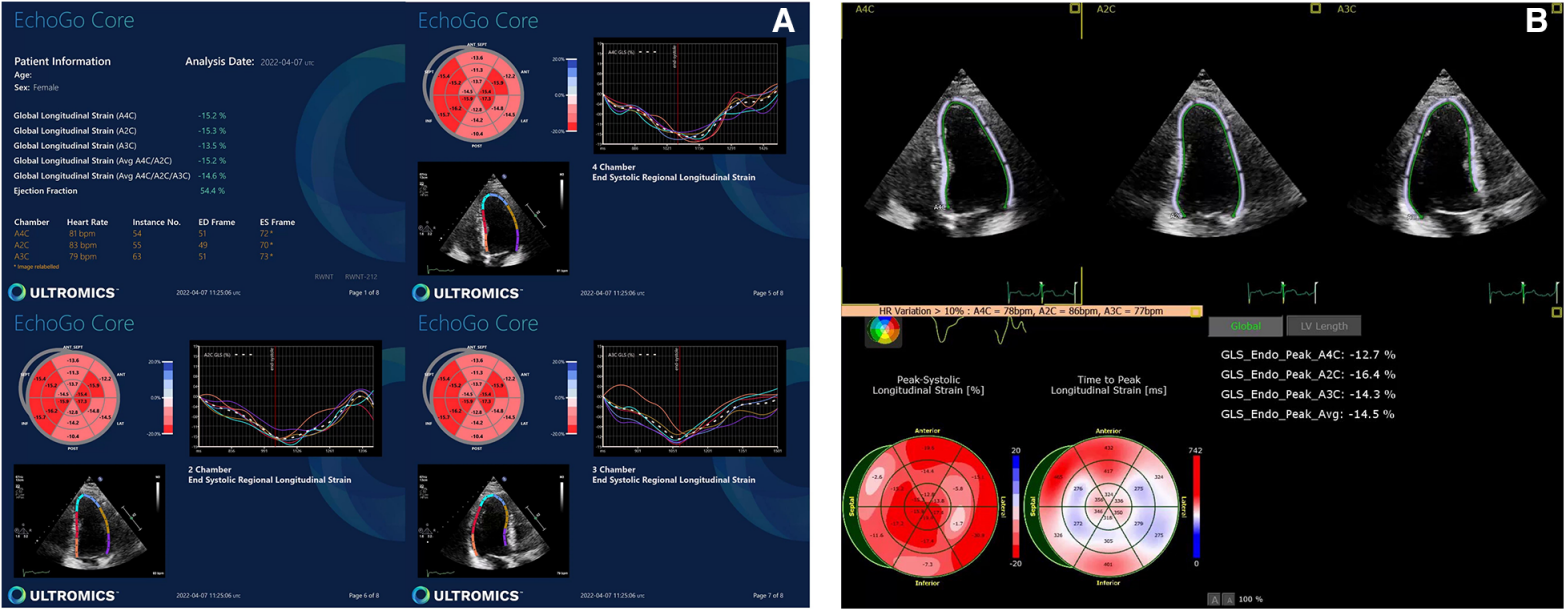
Abnormal GLS data yielded by (**A**) AI-based and (**B**) conventional semi-automated strain analysis.

**Figure 3:**
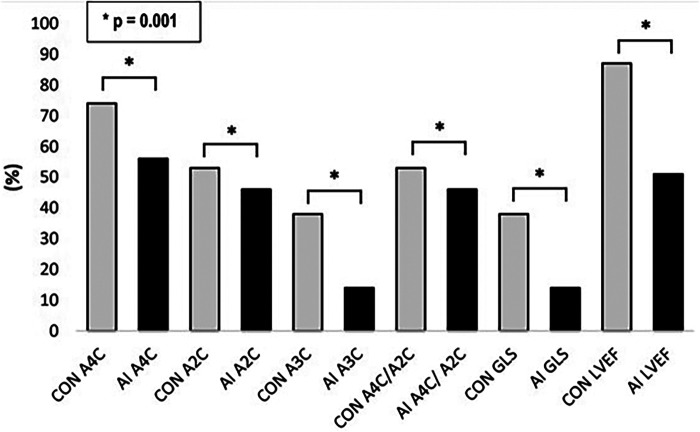
Feasibility of AI-based versus conventional semi-automated strain analysis and LVEF in the standard apical views.

AI-generated GLS and LVEF values were acquired in 14% and 51% of all studies, respectively. Representative examples of normal and abnormal GLS studies analysed by AI-generated and conventional assessment are shown in Figures 1, 2 respectively. The rate of success in obtaining strain results using AI vs. conventional methods for the three standard apical views were: A4C, 56% vs. 74%; A3C, 14% vs. 38%; A2C, 46% vs. 53%, respectively (Figure 3).

Technical failure to derive strain from the A3C was therefore the main reason for the low rate of success in obtaining AI-generated GLS (ANOVA *p* = 0.028). Whilst the success rate of deriving longitudinal strain from the A3C via the conventional method was also low, the failure rate was superior to that of AI. Factors contributing to suboptimal image quality, particularly affecting the A3C, included challenging body composition, tachyarrhythmias, ectopy, limited rib space and previous mastectomy.

### GLS and LVEF using AI vs. conventional assessment

**Figure 4:**
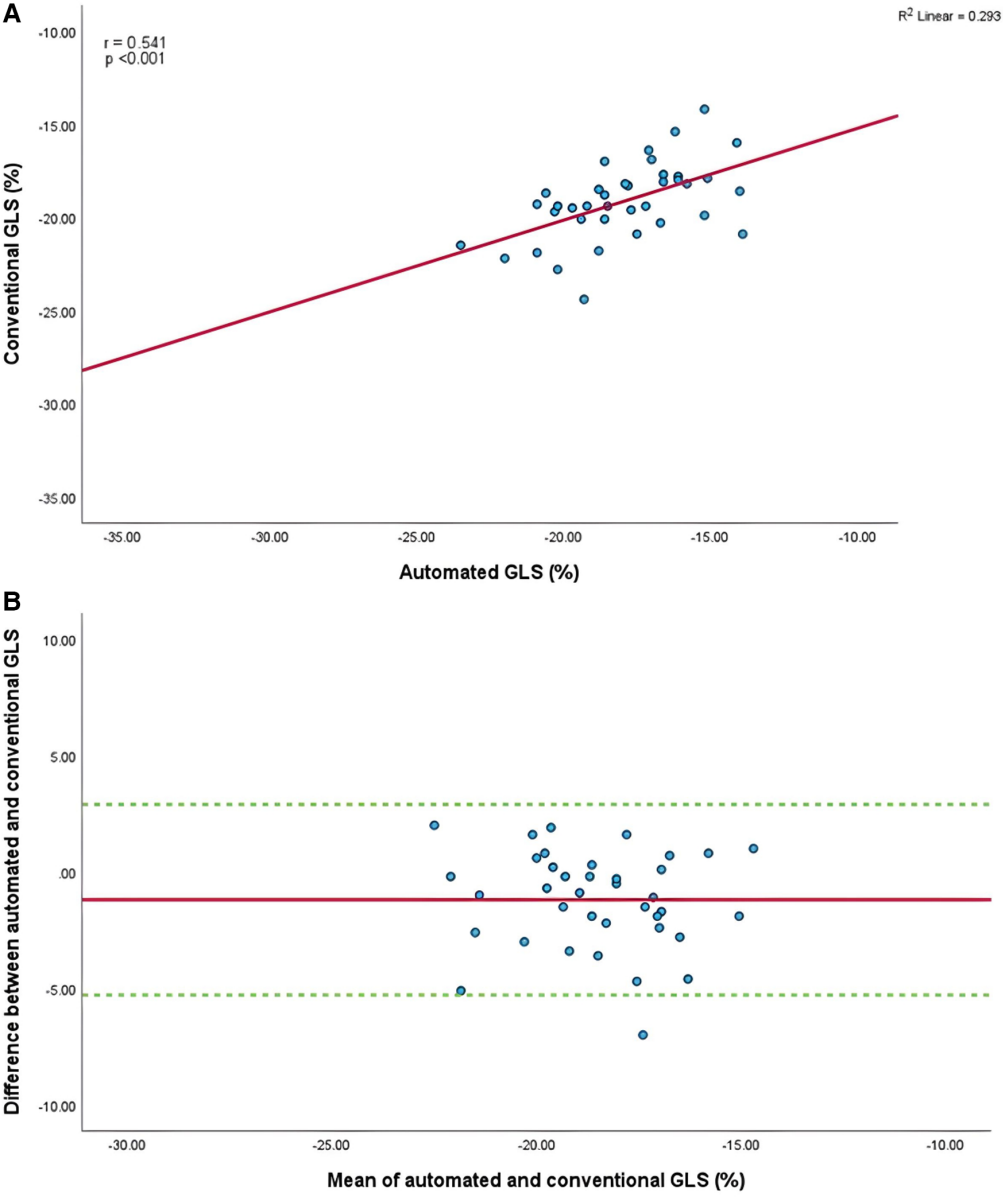
(**A**) Correlation between conventional and AI-automated global longitudinal strain. (**B**) Bland-Altman plot of conventional and AI-automated global longitudinal strain.

**Figure 5:**
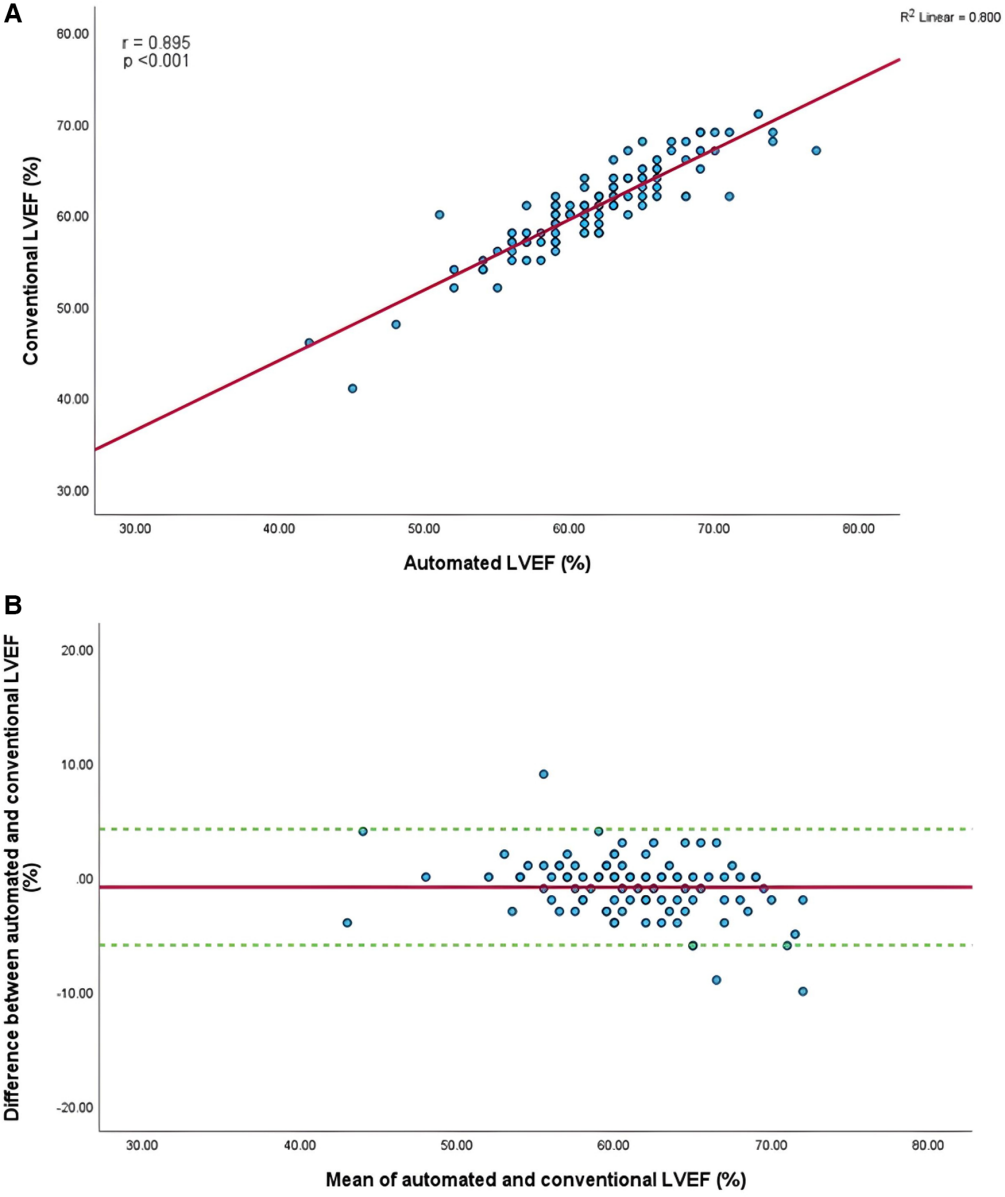
(**A**) Correlation between conventional and AI-automated left ventricular ejection fraction. (**B**) Bland-Altman plot of conventional and AI-automated left ventricular ejection fraction.

Mean GLS in whole cohort was −17.9 ± 2.2% (AI) vs. −19.1 ± 2.0% (conventional). Mean LVEF in the whole cohort was 61.6 ± 5.7% (AI) vs. 60.7 ± 4.9% (conventional). Linear regression and Bland-Altman analysis for GLS revealed moderate correlation (*r* = 0.541, *p* < 0.001) and disagreement (mean bias −1.2%, 95% CI: −5.2% to 2.8%; Figures 4A,B). In contrast, LVEF showed strong correlation (*r* = 0.895, *p* < .001) with small biases (Figures 5A,B).

### Comparison between strain at individual apical views using AI vs. conventional assessment

**Figure 6:**
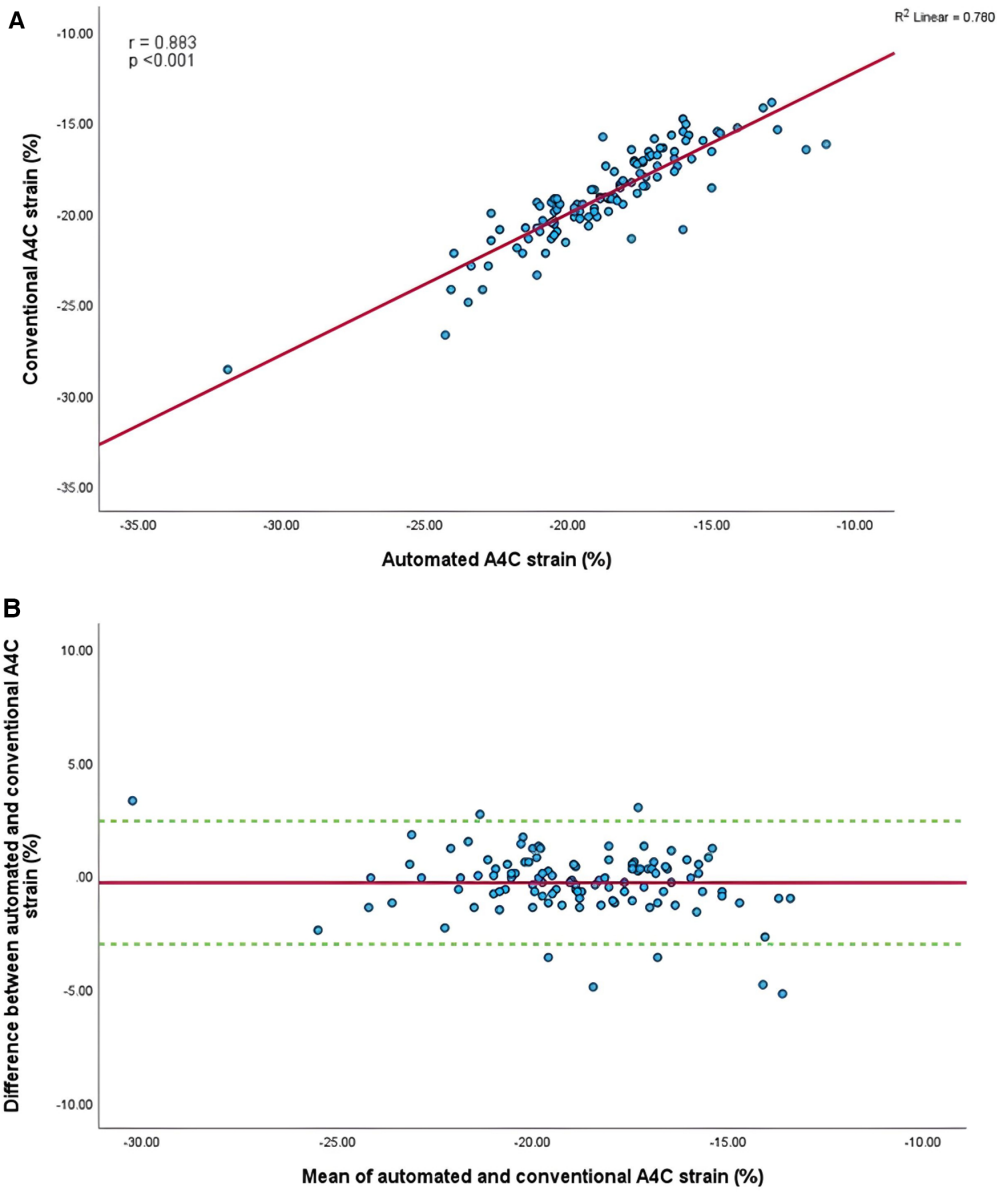
(**A**) Correlation between conventional and AI-automated strain in apical-four chamber view. (**B**) Bland-Altman plot of conventional and AI-automated strain in apical-four chamber view.

**Figure 7:**
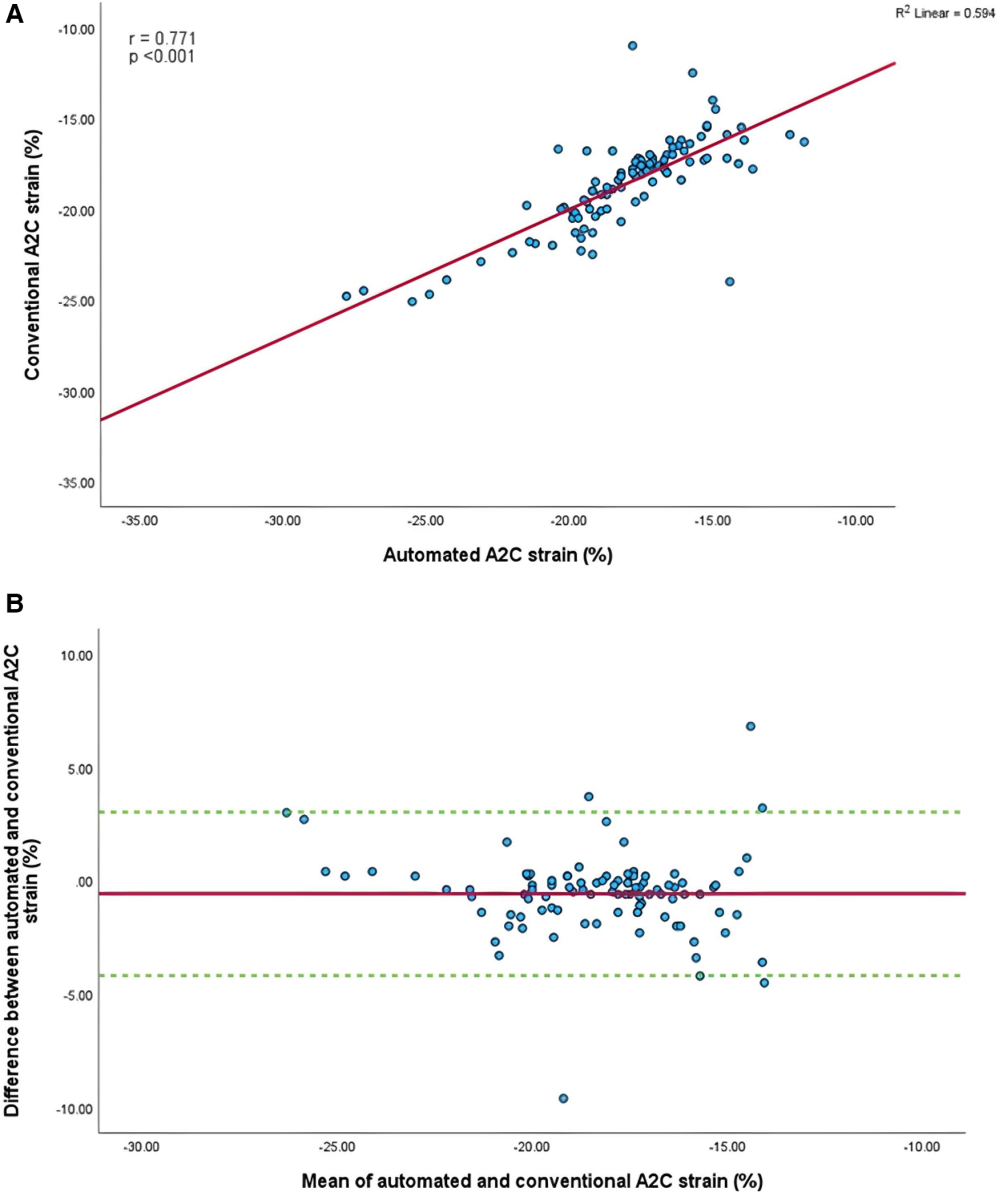
(**A**) Correlation between conventional and AI-automated strain in apical-two chamber view. (**B**) Bland-Altman plot of conventional and AI-automated strain in apical-two chamber view.

**Figure 8:**
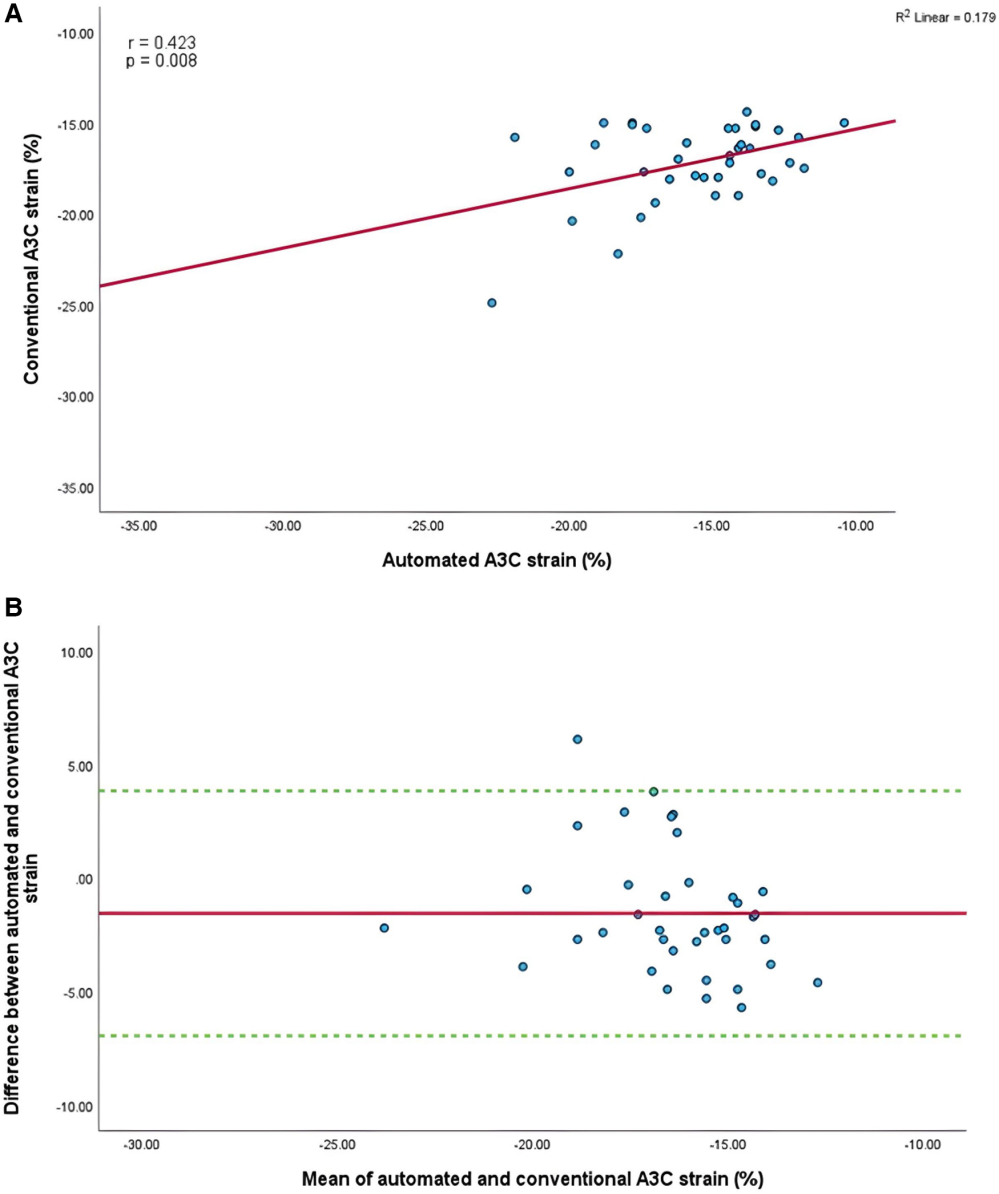
(**A**) Correlation between conventional and AI-automated strain in apical-three chamber view. (**B**) Bland-Altman plot of conventional and AI-automated strain in apical-three chamber view.

**Figure 9:**
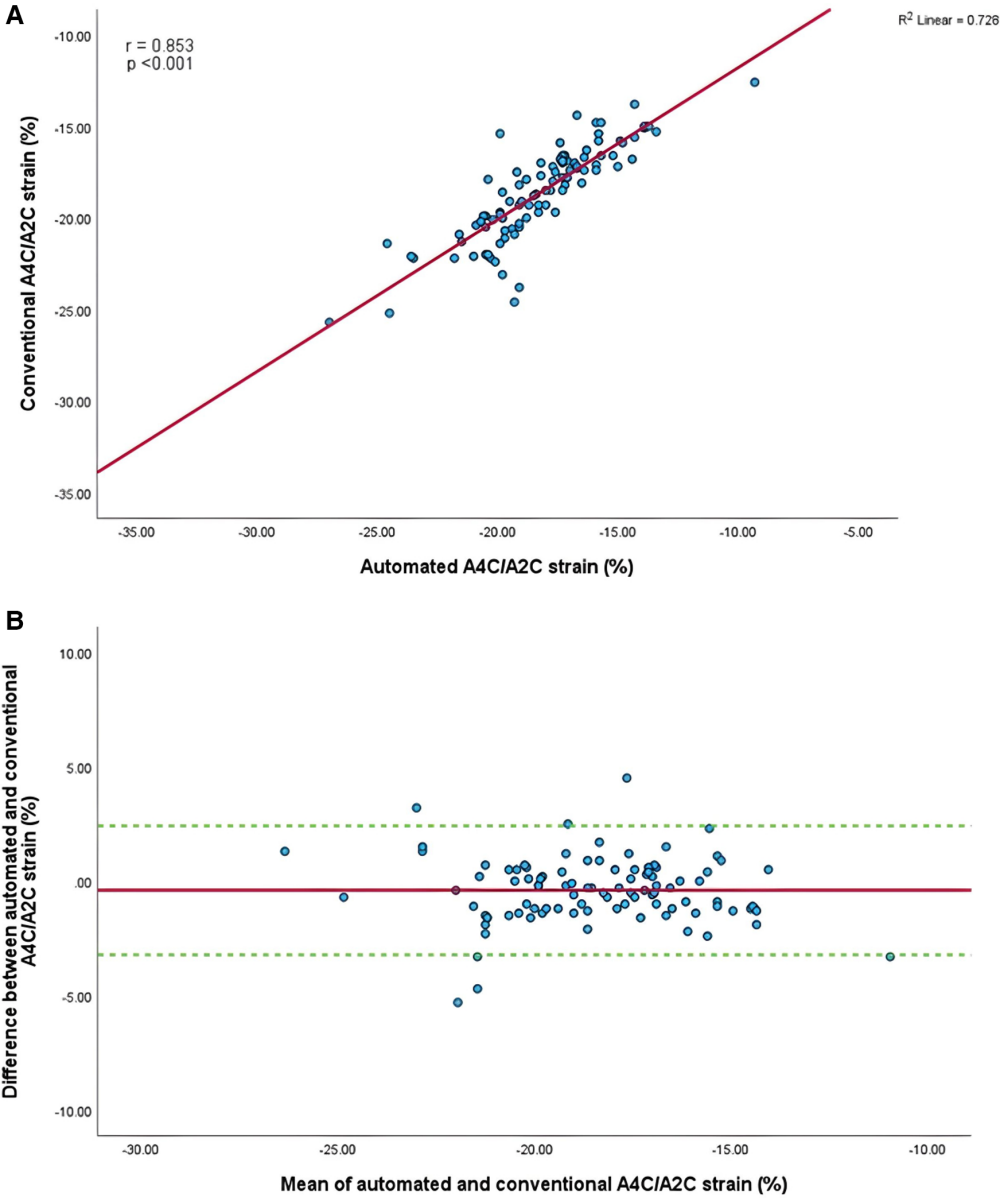
(**A**) Correlation between conventional and AI-automated strain in apical-four/-two chamber view. (**B**) Bland-Altman plot of conventional and AI-automated strain in apical-four/-two chamber view.

Mean longitudinal strain values from specific apical views were −18.7 ± 2.9% and −19.0 ± 2.6% (A4C) (Figures 6A,B), −18.1 ± 2.8% and −18.6 ± 2.6% (A2C) (Figures 7A,B), −15.7 ± 2.6% and −16.6 ± 1.6% (A3C) (Figures 8A,B), and −18.2 ± 2.7% and −18.6 ± 2.6% for the AI method and the conventional method, respectively. A strong correlation and agreement was demonstrated in the A4C (*r* = 0.883, *p* < .001, 95% CI: −3.0% to 2.4%) and A4C/A2C (measurable values achieved from both A4C and A2C views within a given study) strain (*r* = 0.853, *p* < .001, 95% CI: −3.2% to 2.4%) views for strain between AI-automated and conventional methods (Figures 9A,B). In comparison, the A2C strain revealed a moderate correlation (*r* = 0.771, *p* < .001). The weakest correlation (*r* = 0.423, *p* = 0.008) and widest limits of agreement among each individual apical view were observed in the A3C view.

### Temporal changes in GLS and LVEF between AI vs. conventional assessments during surveillance

**Figure 10:**
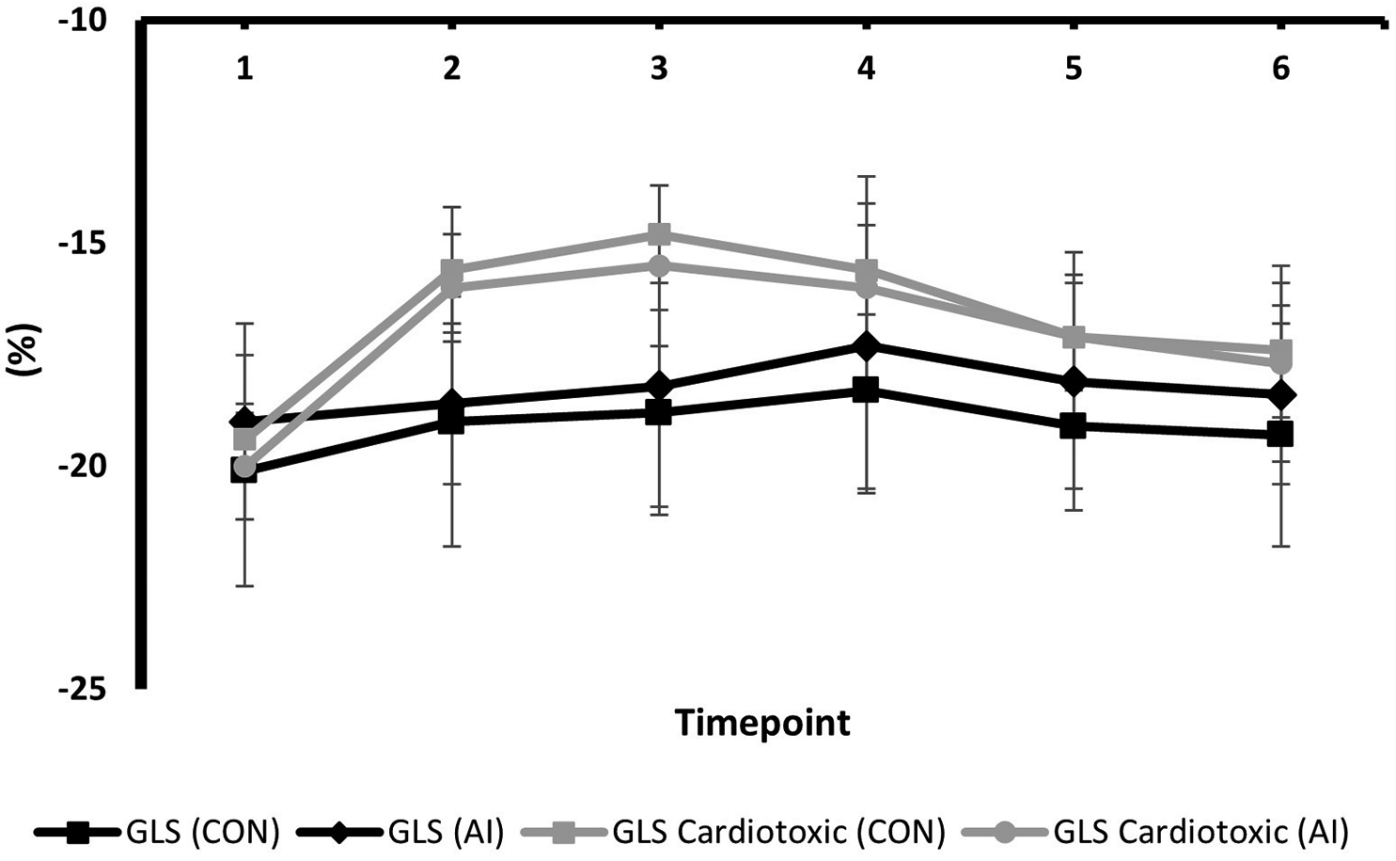
Mean values and standard deviation of conventional GLS and AI-automated GLS at individual timepoints during trastuzumab therapy in the study population.

**Figure 11:**
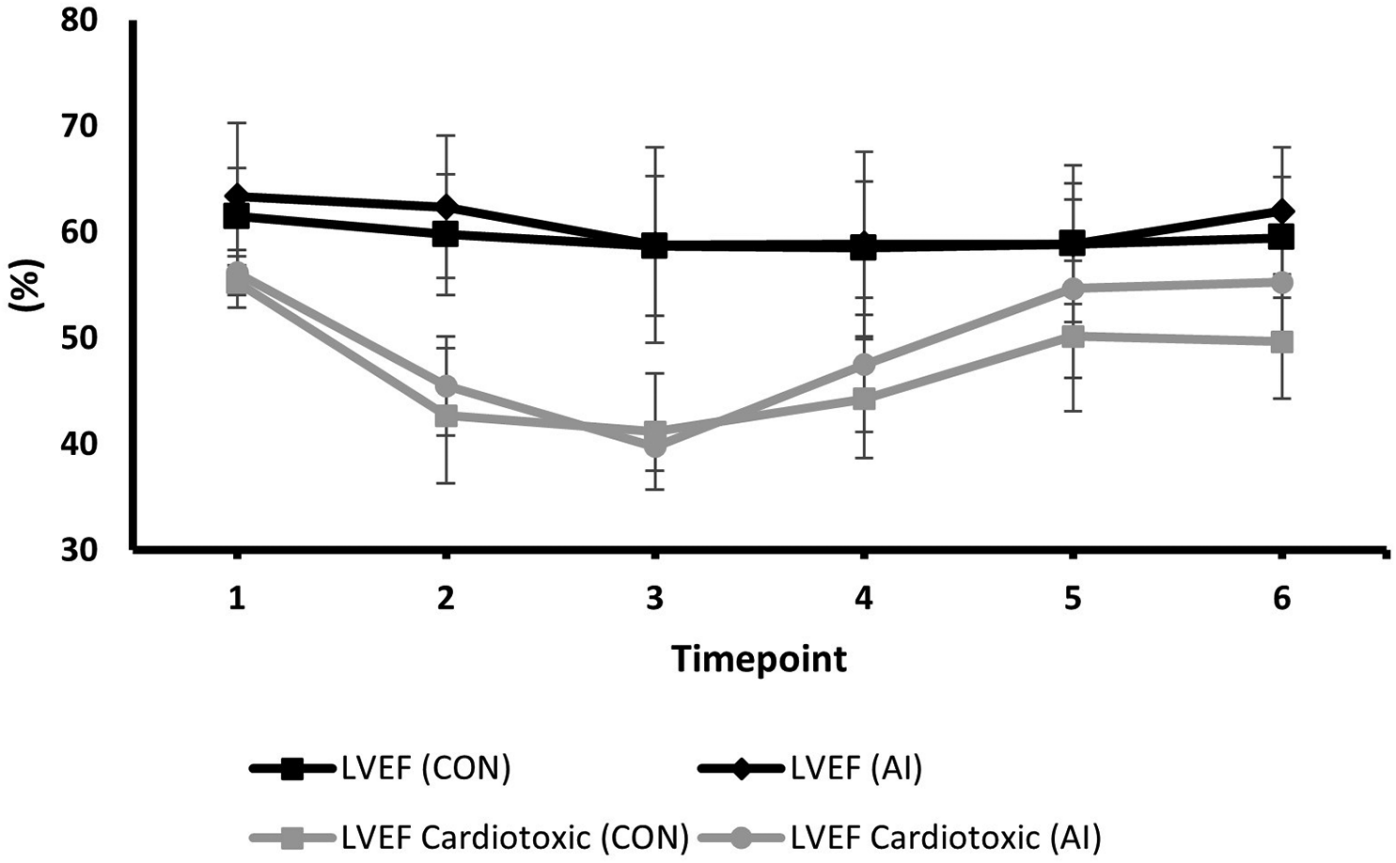
Mean values and standard deviation of conventional LVEF and AI-automated LVEF at individual timepoints during trastuzumab therapy in the study population.

Serial changes in strain and LVEF during TTE surveillance are summarised in Table 2. Statistical differences between the conventional and AI-automated methods at each time point are illustrated in Table 3 using the independent sample *t*-test. Conventional and AI-automated values followed a similar temporal pattern in patients receiving trastuzumab therapy for both GLS and LVEF irrespective of the cardiotoxic cohort or the total study population (Figures 10, 11). At 3 months (T1), both conventional and automated method demonstrated a reduction in GLS and LVEF compared to baseline measurements (T0). By 6 months (T2), further reduction in LV function was observed to a similar degree by both methods. The GLS and LVEF were seen to be lowest at 9 months (T3) from the initiation of trastuzumab therapy. The AI-automated GLS values were consistently more negative lower at each timepoint compared to the conventional method (Table 3 and Figure 11). The LVEF values at timepoint 3 to 5 were almost identical by both methods although a higher degree of variation was observed from the AI-automated method (T3: 58.9 ± 8.7%, *p* = 0.422; T4: 58.9 ± 7.4, *p* = 0.638; T5: 62 ± 6.0, *p* = 0.038). At 12- (T4) and 15-months (T5), AI-automated values demonstrated improvements in GLS and LVEF. Similar trends were observed from the conventional method although the degree of improvement is shown to be smaller in LVEF at 15-months. There were no significant differences observed between the AI-automated and conventional methods for GLS. For LVEF, there was a significantly lower LVEF from the conventional method (59.5 ± 5.7% vs. 62 ± 6.0%, *p *= 0.038). Based on the GLS and LVEF criteria (11), six patients developed cardiotoxicity; this number was considered too small to allow statistical sub-analysis. Nevertheless, the limited cases have highlighted the ability for AI-automated analysis in detecting left ventricular changes among the cardiotoxic cohort.

**Table 2:** Mean values and standard deviation of conventional GLS and AI-automated GLS at individual timepoints during trastuzumab therapy.

	T0	T1	T2	T3	T4	T5
GLS (CON)	−20.1 ± 2.6	−19.0 ± 2.8	−18.8 ± 2.3	−18.3 ± 2.3	−19.1 ± 1.9	−19.3 ± 2.5
GLS (AI)	−19.0 ± 2.2	−18.6 ± 1.8	−18.2 ± 2.7	−17.3 ± 3.2	−18.1 ± 2.4	−18.4 ± 2.0
A4C (CON)	−20.0 ± 3.1	−19.0 ± 3.2	−18.9 ± 2.7	−18.6 ± 3.0	−19.1 ± 2.3	−19.4 ± 2.6
A4C(AI)	−19.8 ± 3.9	−19.4 ± 4.0	−18.5 ± 3.5	−18.8 ± 3.7	−19.3 ± 3.1	−19.1 ± 3.1
A2C CON)	−20.8 ± 2.9	−19.2 ± 3.7	−18.5 ± 4.1	−18.8 ± 2.7	−19.6 ± 2.4	−20.0 ± 3.5
A2C (AI)	−20.7 ± 4.2	−19.2 ± 4.2	−18.4 ± 3.9	−18.1 ± 3.2	−19.1 ± 4.4	−19.7 ± 4.1
A3C CON)	−19.6 ± 3.1	−18.8 ± 3.8	−18.8 ± 2.5	−18.3 ± 2.9	−19.3 ± 2.8	−18.8 ± 3.3
A3C (AI)	−15.3 ± 3.0	−15.5 ± 2.0	−15.7 ± 3.5	−13.9 ± 3.7	−15.5 ± 2.8	−14.9 ± 3.7
LVEF (CON)	61.5 ± 4.6	59.8 ± 5.7	58.7 ± 6.6	58.5 ± 6.3	58.9 ± 5.7	59.5 ± 5.7
LVEF (AI)	63.4 ± 6.9	62.4 ± 6.7	58.8 ± 9.2	58.9 ± 8.7	58.9 ± 7.4	62 ± 6.0

Data are expressed as mean ± standard deviation.

AI, artificial intelligence; A4C, apical-four chamber; A2C, apical-two chamber; A3C, apical-three chamber; CON, conventional; GLS, global longitudinal strain; LVEF, left ventricular ejection fraction.

**Table 3:** AI-Automated and conventional global longitudinal strain and left ventricular ejection fraction at each timepoint.

	Method of assessment		Pearson correlation coefficient	*p*-value
	Conventional (%)	Automated (%)		
GLS (T0)	−20.1 ± 2.6	−19.0 ± 2.2	0.835	>0.001
GLS (T1)	−19.0 ± 2.8	−18.6 ± 1.8	0.856	>0.001
GLS (T2)	−18.8 ± 2.3	−18.2 ± 2.7	0.779	0.004
GLS (T3)	−18.3 ± 2.3	−17.3 ± 3.2	0.761	0.020
GLS (T4)	−19.1 ± 1.9	−18.1 ± 2.4	0.782	0.017
GLS (T5)	−19.3 ± 2.5	−18.4 ± 2.0	0.727	0.023
LVEF (T0)	61.5 ± 4.6	63.4 ± 6.9	0.811	0.019
LVEF (T1)	59.8 ± 5.7	62.4 ± 6.7	0.715	0.031
LVEF (T2)	58.7 ± 6.6	58.8 ± 9.2	0.866	>0.001
LVEF (T3)	58.5 ± 6.3	58.9 ± 8.7	0.838	0.011
LVEF (T4)	58.9 ± 5.7	58.9 ± 7.4	0.844	0.006
LVEF (T5)	59.5 ± 5.7	62 ± 6.0	0.613	0.032

Data are expressed as mean ± standard deviation.

GLS, global longitudinal strain; LVEF, left ventricular ejection fraction.

## Discussion

In this real-world service evaluation and audit of the assessment of left ventricular ejection fraction and strain in a cohort of patients receiving trastuzumab chemotherapy, we assessed whether an AI-automated solution to LV systolic function is a feasible and reliable methodology compared to conventional analysis. The main findings are firstly, that GLS and LVEF quantification obtained from AI-automated assessment showed moderate to strong correlation compared to conventional methods. Secondly, AI-generated GLS and LVEF values compared reasonably well with conventional methods, demonstrating a similar temporal pattern throughout the echocardiographic surveillance. Thirdly, the apical-three chamber view demonstrated the lowest correlation and revealed to be least successful for acquisition of GLS and LVEF. Finally, compared to conventional methodology, AI-automated analysis has a significantly lower feasibility rate, demonstrating a success rate of 14% (GLS) and 51% (LVEF).

### Clinical demand and relevance

While the introduction of speckle tracking has provided exciting opportunities in the field of cardiac imaging, its clinical application is rendered meritless if performed by unexperienced or suboptimally trained practitioners. Like any echocardiographic technique, there is a steep learning curve with performing and interpreting echocardiograms (12). Interpretation of echocardiographic studies is demanding and this can limit workflow particularly among smaller centres with fewer trained echocardiographers. The application of AI echocardiography may potentially address these challenges by utilising an AI-based analysis of LV strain.

There is emerging data suggesting that a fully automated AI assessment could potentially reduce post-processing time with high reproducibility and reduced risk imposed by human-software interaction. However, in the presence of significant knowledge gaps the technology may fall short of this potential. Presently, semi-automated assessments are in clinical use and accepted as a standard, feasible method for LV strain assessment, supported by evidence from numerous studies have supported the use of these methods (13–16). However, the human-software interaction is such that the current semi-automated approach yields values that are highly influenced by the level of experience and training of the sonographer.

Furthermore, research to date has rarely explored the application of AI-automated assessment in cancer therapy-related cardiac dysfunction but instead has largely focused on ischaemia-related cardiac abnormalities. Given that cancer therapy-induced heart failure carries a worse prognosis compared to heart failure related to other causes (17), the need for accurate and frequent echocardiographic surveillance is clear and of paramount importance. It follows that there is a clinical need for research into AI-automated detection of subclinical changes in cardiac function to accurately, reliable and rapidly detect changes earlier in the disease process. To the best of our knowledge, this is the first real-world evaluation of such an approach to validate and explore the clinical feasibility of AI-automated LV assessment in this patient cohort throughout the surveillance period.

### The feasibility and accuracy of automated GLS and LVEF

The present findings reveal that the current version of AI-automated GLS possess some limitations in feasibility, achieving successful acquisition of GLS in only 14% of all studies. The higher rate of success demonstrated from conventional methods (38%) suggests either that the AI-automated approach is inferior to the semi-automated approach or that the semi-automated approach is overly generous in the studies to which it is applied. The unifying consideration here is that of a threshold for acceptability for an echo study to be amenable to either of the assessment methods. We speculate that the two approaches accept image qualities of different levels. Standardising this threshold is not necessarily a straight-forward proposition as even with a group of selected studies, the AI-automated system is using different approaches to strain assessment than in the semi-automated system.

In either analysis approach, the acquisition of GLS requires the strain values of three individual apical views. The present study found that the A3C view was the most frequently limiting view followed by the A2C (46%) in preventing a GLS assessment. These findings are in keeping with a study by Kawakami et al. which examined the automated tracking quality in each individual LV segments (14). The study found that the LV segments in these in these views are often associated with considerably poorer automated tracking compared to segments in the A4C view.

In contrast to previous studies that excluded echo studies where image quality were deemed substandard (14), the present analysis did not exclude these patients and is therefore relevant to real-world clinical practice. All oncology patients that were administered trastuzumab and underwent echo surveillance were included to minimise selection bias and reflect real-world patient cohorts, including known imaging challenges often specific to cardio-oncology patients such as radiotherapy, breast reconstruction surgeries, mastectomy and breast implantation (18). This might explain the lower rate of successful acquisition compared to previous trials as the availability of diagnostic quality images are reduced. Conversely, the possibility for over-analysis in potentially non-feasible images should not be excluded. The likelihood of the operator repeatedly adjusting the region of interest in the presence of limited or absence of endocardial border definition to “inaccurately” create a GLS value that is consistent with visual assessment is not uncommon and ought to be considered.

Previous validation studies (8, 9, 14, 19) comparing AI-automated and conventional methods have reported good feasibility and correlation values, often in patient groups with ischaemia-related heart diseases and other pathologies unrelated to chemotherapy. In the setting of cardio-oncology, our results are in line with previously reported evidence which demonstrated a reasonable correlation between AI-derived GLS and LVEF values to the conventional method, suggesting that there were no considerable differences between method of assessments.

Although our reported values were lower compared to the literature, this may be influenced by the preselection of subjects with segments suited for assessment in previous studies. Our findings also demonstrated that serial monitoring of trastuzumab-treated oncology patients with AI-assisted technology to detect subtle changes in LVEF and GLS may be done with similar certainty to conventional assessment with the values generated from both methods being largely similar.

A significant difference in LVEF was observed at one timepoint although this may be attributed to smaller sample size at the final follow-up. Further work will be required to assess longitudinal echocardiographic trends in addition to correlation between AI-automated and conventional analyses, and there may be systematic differences in absolute values whether related to the vendor or system used.

In the cardiotoxic cohort, while the sample size was small, both methods demonstrated a similar temporal trend highlighting the potential for AI-automated methods to reliability detect LV functional deterioration. Such findings suggest that AI-automated LV assessments represent a valuable method of serial echocardiographic monitoring in longitudinal patient care and can build a case for future prospective studies in this area.

### Study limitations

There are a few potential limitations associated with the present analysis that deserves to be mentioned. First, we only studied patients in sinus rhythm, thus data could not be extrapolated from patients with irregular heart rhythms. Additionally, our study included a relatively small sample size. Despite this, our patient cohort included all patients during the study period to reflect a real-world clinical setting and is the first to study functional changes in this specific patient cohort, thereby providing valuable insight into the application of AI-automated analysis in serial echocardiographic studies in trastuzumab-treated patients. Our report and early insights thereby provide a basis for future studies to expand upon. Second, the potential vendor differences in AI-imaging software for strain and LVEF analysis due to differences in AI-algorithms should be noted. Third, is the lack of gold standard reference to compare our strain and EF measurements. However, the primary objective was to determine the level of correlation between AI-automated and conventional methods thus identifying the “true” reference value is of lesser significance. We therefore used the current clinically accepted semi-automated approach as the comparator. Finally, the analysis was conducted retrospectively which meant that it suffered from the inherent limitations of a restrospective study design. Nevertheless, this report describes a straightforward comparision of imaging as opposed to patient outcomes, thus selection bias is of lesser relevance.

### Future research directions

With increasing echocardiographic demands surpassing clinical capacity in the face of a shortage of echocardiographers, there is now an urgent need for the active incorporation of AI guided technology to assist, or potentially substitute the need for operator input into analysis of advanced echocardiographic techniques. Consequently, software solutions must possess the accuracy to where it could be confidently applied irrespective of the GLS experience of the operator. There are a number of challenges in the widespread clinical implementation of AI echocardiography, none of which are considered insurmountable.

The future appears positive for the application of AI in echocardiography and significant advances are anticipated to address the current knowledge gaps. Future work should explore whether: (1) AI-based assessment is superior to less experienced humans, (2) image rejection threshold appropriateness, (3) accuracy and reproducibility of automated, semi-automated and manually generated data, and (4) improvements in post-processing time and overall workflow on echocardiography services.

## Conclusions

Despite enthusiasm for the application of AI technology in healthcare, it is yet to be widely embraced in the echocardiographic community. Due to significant limitations and knowledge gaps in automation, AI technology in echocardiography remains premature for clinical use if adopted completely independent of operator intervention. Instead, at present, it could be a useful unbiased “second opinion” for “experienced” practitioners. Our analysis is supportive of prospective studies into the utility and application of AI-based analysis of heart function by echocardiography in patients receiving potentially cardiotoxic chemotherapy.

## Data Availability

The original contributions presented in the study are included in the article/Supplementary Material, further inquiries can be directed to the corresponding author.
